# Site-Directed Immobilization of an Engineered Bone Morphogenetic Protein 2 (BMP2) Variant to Collagen-Based Microspheres Induces Bone Formation In Vivo

**DOI:** 10.3390/ijms23073928

**Published:** 2022-04-01

**Authors:** Claudia Siverino, Shorouk Fahmy-Garcia, Didem Mumcuoglu, Heike Oberwinkler, Markus Muehlemann, Thomas Mueller, Eric Farrell, Gerjo J. V. M. van Osch, Joachim Nickel

**Affiliations:** 1 Department of Tissue Engineering and Regenerative Medicine, University Hospital Wuerzburg, 97070 Wuerzburg, Germany; claudia.siverino@aofoundation.org (C.S.); heike.oberwinkler@uni-wuerzburg.de (H.O.); markus.muehlemann@ymail.com (M.M.); 2 Department of Orthopaedics and Sports Medicine, Erasmus MC, University Medical Center, 3015 GD Rotterdam, The Netherlands; s.fahmygarcia@erasmusmc.nl (S.F.-G.); didemmumcuoglu@gmail.com (D.M.); g.vanosch@erasmusmc.nl (G.J.V.M.v.O.); 3 Department of Internal Medicine, Erasmus MC, University Medical Center, 3015 GD Rotterdam, The Netherlands; 4 Department of Oral and Maxillofacial Surgery, Erasmus MC, University Medical Center, 3015 GD Rotterdam, The Netherlands; e.farrell@erasmusmc.nl; 5 Fujifilm Manufacturing Europe B.V., 5047 TK Tilburg, The Netherlands; 6 Department for Molecular Plant Physiology and Biophysics, Julius-von-Sachs Institute of the University Wuerzburg, 97082 Wuerzburg, Germany; mueller@biozentrum.uni-wuerzburg.de; 7 Department of Otorhinolaryngology, Erasmus MC, University Medical Center, 3015 GD Rotterdam, The Netherlands; 8 Fraunhofer ISC, Translational Center RT, 97070 Wuerzburg, Germany

**Keywords:** bone morphogenetic protein 2 (BMP2), bone regeneration, covalent coupling, subcutaneous animal model

## Abstract

For the treatment of large bone defects, the commonly used technique of autologous bone grafting presents several drawbacks and limitations. With the discovery of the bone-inducing capabilities of bone morphogenetic protein 2 (BMP2), several delivery techniques were developed and translated to clinical applications. Implantation of scaffolds containing adsorbed BMP2 showed promising results. However, off-label use of this protein-scaffold combination caused severe complications due to an uncontrolled release of the growth factor, which has to be applied in supraphysiological doses in order to induce bone formation. Here, we propose an alternative strategy that focuses on the covalent immobilization of an engineered BMP2 variant to biocompatible scaffolds. The new BMP2 variant harbors an artificial amino acid with a specific functional group, allowing a site-directed covalent scaffold functionalization. The introduced artificial amino acid does not alter BMP2′s bioactivity in vitro. When applied in vivo, the covalently coupled BMP2 variant induces the formation of bone tissue characterized by a structurally different morphology compared to that induced by the same scaffold containing ab-/adsorbed wild-type BMP2. Our results clearly show that this innovative technique comprises translational potential for the development of novel osteoinductive materials, improving safety for patients and reducing costs.

## 1. Introduction

The management of critical-size bone defects remains a challenging medical intervention associated with a high economic burden for the society [1,2]. Currently, bone graft substitutes derive from the need to find an alternative to the historically used technique of autologous bone grafting [3]. However, autologous bone transplantation is time consuming and associated with increased postoperative pain and complications, such as injury to sensory or motor nerves, hematoma and infection [4].

The necessity to improve patients’ health and to minimize adverse events connected to the use of autologous bone grafts resulted in the development of a readily available graft material supplemented with a growth factor, e.g., bone morphogenetic protein 2 (BMP2). The short half-life of BMP2 when systemically administered was reported to be 16 min in rat and 7 min in non-human primates, leading to the investigation and application of different protein delivery methods [5,6]. Indeed, since its discovery and its approval by the FDA, BMP2-loaded scaffolds have been intensively used in the clinics for single-level anterior lumbar interbody fusion (ALIF), tibial non-unions and oral maxillofacial reconstructions [7,8,9]. However, use of the BMP2-loaded scaffolds induced several side effects, most likely connected to the supraphysiological amounts of protein used. The initial burst release and the accompanying continuous loss of protein are responsible for the high costs and the large amount of BMP2 needed to induce bone formation at the implantation site [10]. Therefore, in recent decades, researchers have focused on developing improved bone grafting strategies that, other than complying with the safety aspects, including purity, immunogenicity and biocompatibility, focus on the biomaterial’s intrinsic activity and/or on the growth factors [11].

The scaffold’s basic requirements are biocompatibility and biodegradability to allow cell infiltration and de novo formation of blood vessels into the newly formed bone [12,13]. Additionally, the scaffold characteristics are crucial to improve protein delivery. The material composition influences the protein release kinetics and needs to shield the BMP2 from degradation and thus maintain its bioactivity. BMP2 is usually ab-/adsorbed to the scaffold, causing a 50% loss of protein after a few days in vivo [14]. Following an ideal release kinetic, the protein should be released in a spatiotemporally controlled manner to promote new bone formation at the treatment site. Therefore, instead of just ad-/absorbing the protein, several other studies focused on different strategies for protein immobilization [15,16,17]. The insertion of different affinity tags to the protein sequence has been intensively investigated [18]. This method entails highly specific interactions, mild coupling conditions and the availability of a broad variety of different commercially available affinity tag systems (poly-arginine or poly-histidine, FLAGs, Myc and many others). However, the introduction of longer tags is limited to specific restricted positions of the protein, mainly to the N-terminus. The immobilization by tags located at the ligand’s N-terminus results in a poor orientation of the protein toward cell surface receptors and thus might induce a partial or a total loss of its bioactivity due to structural instabilities or blockage of the receptor binding sites [19]. Alternatively, BMP2 was covalently coupled utilizing functional groups within the protein, such as amino or carboxyl groups [20,21,22,23,24,25]. However, since these reactions cannot be tightly controlled, the use of these techniques results in a random, not directed immobilization of the protein, thus creating scaffolds with variable osteogenic properties [26,27,28].

The use of site-directed immobilization techniques would overcome all limitations described for the previous approaches. The introduction of artificial amino acids in specific positions of the protein sequence already occurs during protein synthesis in modified bacterial expression systems [29]. This technique increases the variety of functionalized proteins useful to investigate protein structure and function and cellular mechanisms [30,31,32]. In contrast to affinity tags, these artificial amino acids can be introduced in different positions within the primary protein structure, which can, however, impact function and bioactivity [33]. In a previous study, a BMP2 variant harboring an artificial Propargyl-l-lysine (Plk) residue was designed to test covalent and site-directed coupling to beads by copper-catalyzed azide–alkyne cycloaddition (CuAAC) [34]. We could show that this BMP2 variant retained its bioactivity in cell-based assay upon coupling to different types of beads. The coupling procedure, described by Tabisz et al., used a classic click chemistry protocol using Cu(I) as catalyst [34]. This reaction is known to provoke conformational, structural and biological changes and, indeed, in our previous study, also appeared to compromise the protein’s structure stability [34,35,36]. In that study, the introduced artificial amino acid replaced the third amino acid (lysin3) at the BMP2 N-terminus. This position was selected, since from our own experience, it best guaranteed proper refolding and purification of the expressed protein. Furthermore, it would enable an acceptable orientation of the immobilized ligand toward cell receptors due to the flexibility of the ligand’s N-terminus. However, after coupling BMP2 K3Plk to bulky structures (e.g., beads), the initiation of BMP signaling was hampered, most likely due to steric hindrance.

To prevent these limitations, we propose here a new BMP2 variant with an artificial H-L-Lys(EO-N_3_)-OH introduced at position 83 of each chain of BMP2 being coupled to collagen-based beads by a copper-free click chemistry approach [37]. The selected position of the artificial amino acid within the BMP2 structure enables an optimal orientation toward BMP’s cellular receptors, even if the ligand is immobilized to bulky surfaces. With this work, we suggest a promising approach to produce reproducible osteogenic surfaces requiring much lower protein amounts, thus resulting in lower manufacturing costs and, most importantly, user safety.

## 2. Results

### 2.1. BMP2 WT and BMP2 Azide Bind to BMP Receptors with Comparable Binding Affinities In Vitro

**Figure 1:**
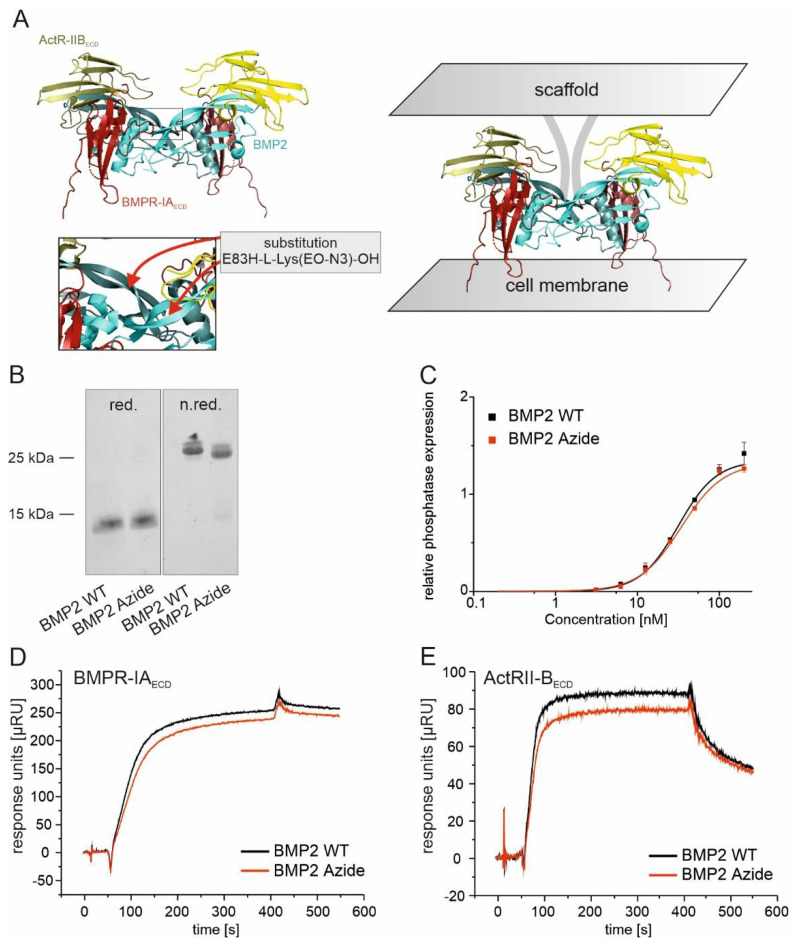
Structural and cell-based analyses of BMP2 Azide compared to the wild type BMP2. (**A**) Ribbon structure of the heterohexameric BMP2:BMPR-IA_ECD_:ActR-IIB_ECD_ complex [33]. Cartoons of the structures were created using the pyMol 3.7 software. The underlying coordinates were provided by the public database RCDS PDB (accession number 2H64). The insert with higher magnification indicates the position of the introduced artificial amino acid (H-L-Lys(EO-N_3_)-OH) within BMP2 (monomers are depicted in light green, the receptor ectodomain of BMPR-IA_ECD_ (red) and ActR-IIB_ECD_ (yellow)). BMP2 Azide being coupled to the scaffold is oriented toward the cell membrane (right panel); (**B**) Coomassie Brilliant Blue staining of BMP2 WT and BMP2 Azide under reducing (left panel) and non-reducing (right panel) conditions; (**C**) The graph shows dose-dependent ALP expression in C2C12 cells induced by BMP2 Azide (red) and BMP2 WT (black). Relative phosphatase expression shows absorption at 405 nm after subtraction of the background absorption at 405 nm; (**D**,**E**) Surface plasmon resonance (SPR) sensorgrams depict the interactions of the indicated ligands with (**D**) BMPR-IA_ECD_ and (**E**) ActR-IIB_ECD_ at 80 nM ligand concentrations. Apparent *K_D_* values (presented in text) were calculated as described in the Materials and Methods section. Statistical analysis for the SPR data was performed using unpaired t-test. No significant difference between the ligands was evaluated.

The ribbon structure depicts the heterohexameric BMP2 WT:BMPR-IA_ECD_:ActR-IIB_ECD_ complex (Figure 1A) [38]. The introduction of the artificial amino acid (H-L-Lys(EO-N_3_)-OH) in a specific position of the ligand (see insert with higher magnification in Figure 1A) allows the covalent immobilization to a scaffold while maintaining the orientation toward the BMP2 receptors. Analysis of the purified BMP2 Azide by SDS-PAGE and Coomassie Brilliant Blue staining revealed, as expected, a single band of ~26 kDa under non-reducing and ~13 kDa under reducing conditions, respectively (Figure 1B). Moreover, both bands showed electrophoretic mobilities similar to those observed for BMP2 WT, which was used as control (Figure 1B). The biological activities expressed as the half-maximal effective concentrations (EC_50_ values) of BMP2 Azide (27.63 ± 5.33 nM) and BMP2 WT (30 ± 5.94 nM) were comparable in ALP assay (Figure 1C) and in agreement with already published data [34]. Surface plasmon resonance (SPR) spectroscopy was used to identify possible changes in receptor binding characteristics in vitro. Both, BMP2 WT and BMP2 Azide showed no differences in binding kinetics to the BMP2 receptors BMPR-IA_ECD_ and ActR-IIB_ECD_ (Figure 1D,E). The calculated mean equilibrium constants *K_D_* (BMP2 Azide: 2.51 × 10^−9^ M, BMP2 WT: 1.90 × 10^−9^ M) for the type I receptor BMPR-IA_ECD_ and for the type II receptor ActR-IIB_ECD_ (BMP2 Azide: 1.16 10^−8^ × M, BMP2 WT: 8.26 × 10^−9^ M) were at comparable levels.

### 2.2. BMP2 Azide Can Be Efficiently and Specifically Coupled to a DBCO Functionalized Fluorophore by a Copper-Free SPAAC Reaction

**Figure 2:**
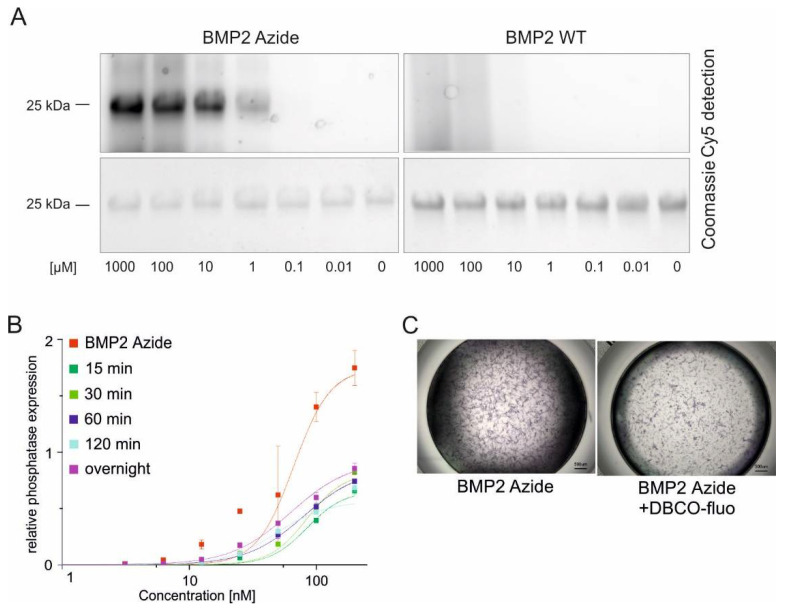
Proof of coupling specificity and maintained bioactivity after a copper-free reaction of BMP2 Azide with a DBCO-functionalized fluorophore. (**A**) BMP2 Azide (20 µM concentration) was coupled to different concentrations of a DBCO-functionalized fluorophore (DBCO-Cy5). Coupling of BMP2 Azide to Cy5-DBCO was detected by fluorescence (upper panel). Gels were stained with Coomassie Brilliant Blue for loading control (lower panel). BMP2 WT was used as negative control; (**B**) Graphs show dose-dependent ligand-induced ALP expression in C2C12 cells by uncoupled BMP2 Azide (red line) and BMP2 Azide (20 µM) coupled to DBCO-functionalized fluorophore (DBCO-PEG_4_-5/6-Texas Red, 100 µM) after being incubated for different reaction times (15 min, 30 min, 60 min, 120 min and overnight). Relative phosphatase expression shows absorption at 405 nm after subtraction of the background absorption at 405 nm. Statistical analyses of the ALP data were performed using two-way ANOVA–Dunnett’s multiple comparisons test. The only significant differences were observed by comparing the ALP activities induced by uncoupled BMP2 Azide and those induced by coupled BMP2 Azide (15, 30, 60 and 120 min of coupling time) at 25 nM concentrations; *p* < 0.05. (**C**) Alkaline phosphatase staining in C2C12 cells incubated with 200 nM uncoupled BMP2 Azide and 200 nM BMP2 Azide coupled to DBCO-PEG_4_-5/6-Texas Red for 2 h. Microscopic images were taken 5 min after addition of NBT/BCIP.

The Azide group in the side chain of the introduced artificial amino acid (H-L-Lys(EO-N_3_)-OH allows a copper-free, covalent coupling by strain-promoted azide–alkyne cycloaddition (SPAAC) with a cyclooctyne group (e.g., dibenzocyclooctyne (DBCO)), demonstrated by a quantitative yield of stable triazoles (Figure S1). Using the same reaction to couple BMP2 Azide to a DBCO functionalized fluorophore (DBCO-Cy5), a concentration-dependent fluorescent signal was observed from a minimum concentration of 1 µM of DBCO-Cy5, while no signal was detected in the negative control using BMP2 WT (Figure 2A, upper panel). This confirmed that coupling occurs specifically via the functional group of the introduced (H-L-Lys(EO-N_3_)-OH) moiety. Equal loading of both proteins was confirmed by Coomassie Brilliant Blue staining (Figure 2A, lower panel). Consequently, the minimal ratio for a stable yield of coupled products was a 20:1 ratio of protein to the DBCO-functionalized fluorophore. However, coupling BMP2 Azide to DBCO-Cy5 resulted in a complete loss of bioactivity after 2 h of incubation (Figure S2). Since protein degradation or bigger structural damages were not observed by Coomassie staining (Figure 2A), we hypothesized that the bulky fluorophore might shield the receptor binding epitopes, thus abolishing the interaction of the ligand with cellular receptors. Therefore, we coupled the BMP2 Azide to a longer spacer containing fluorophore (DBCO-PEG_4_-5/6-Texas Red) and analyzed the maintained bioactivity by ALP assays. Indeed, the coupled protein showed slightly higher EC_50_ values compared to the unreacted control (meaning slightly decreased bioactivities) but comparable among the different reaction times (EC_50_ values, BMP2 Azide 0 min: 66.2 nM, 15 min: 84.5 nM, 30 min: 84.4 nM, 60 min: 79.2 nM, 120 min: 48.7 nM, and overnight: 62.6 nM) (Figure 2B). This observation was also confirmed by reduced ALP staining intensities after exposure to the BMP2 Azide-DBCO-PEG_4_-5/6-Texas Red complex compared to the uncoupled protein (Figure 2C). Both quantitative and qualitative ALP assays show that despite this slight loss of activity, cells exposed to the fluorophore-coupled BMP2 Azide express ALP, suggesting that the protein remains bioactive even after the coupling reaction.

### 2.3. BMP2 Azide Can Be Coupled to Collagen-Based Microspheres with High Efficiency

**Figure 3:**
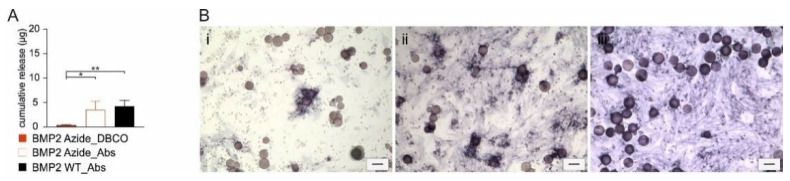
BMP2 Azide is covalently coupled to RCP microspheres with high efficacy and maintains its bioactivity after coupling. An amount of 20 µg of BMP2 Azide reacted with DBCO-functionalized microspheres (_DBCO) or BMP2 Azide and BMP2 WT reacted to unfunctionalized microspheres (_Abs). (**A**) Cumulative release over two weeks after coupling was analyzed by ELISA. Statistical analysis was performed using ordinary one-way ANOVA–Tukey’s test, * *p* = 0.0255 and ** p = 0.0085. (**B**) ALP staining of (**i**) BMP2 Azide coupled to DBCO-functionalized RCP, (**ii**) BMP2 Azide absorbed to RCP or (**iii**) BMP2 WT absorbed to RCP. Scale bar 100 µm.

We then proceeded to couple BMP2 Azide to recombinant collagen-like peptide (RCP) microspheres (Cellnest™, Fujifilm, Tilburg, The Netherlands) that were functionalized using a bivalent NHS-PEG_4_-DBCO linker (Figure S3). As a control, BMP2 Azide or BMP2 WT were incubated with the non-functionalized microspheres, resulting in only ab-/adsorbance of the growth factors. Almost the complete amount of BMP2 Azide that was covalently coupled to the RCP microspheres was retained, while ~30% ad-/absorbed BMP2 Azide or BMP WT was released over 2 weeks in phosphate-buffered saline (PBS) as measured by ELISA (Figure 3A). ALP staining of C2C12 cells confirmed the preserved biological activity of the proteins immobilized or ab-/adsorbed to RCP microspheres (Figure 3B). Notably, only cells in close proximity to RCP microspheres loaded with covalently coupled BMP2 Azide expressed ALP (Figure 3B(i)). In Figure 3B(i), some BMP2 Azide-functionalized microspheres did not induce ALP expression. This might be caused by a non-uniform distribution of BMP2 Azide when reacted to the DBCO-functionalized microspheres. Nevertheless, these results indicate that the coupling reaction prevents diffusion of the growth factor, whereas both ad-/absorbed proteins, BMP2 WT and BMP2 Azide, induced ALP expression also in cells at a further distance (Figure 3B(ii,iii)).

### 2.4. RCP Microspheres Functionalized with the Covalently Coupled BMP2 Azide Induce Ectopic Bone Formation with Similar Bone Volume and Density as Ad/Absorbed BMP2 WT

**Figure 4:**
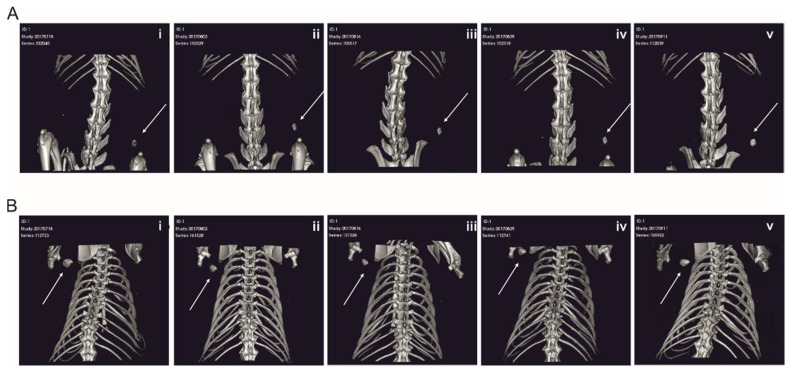
Evaluation of ectopic bone formation in a rat subcutaneous model. Representative micro-CT images of one rat out of the four rats treated with 10 µg (**A**) BMP2 WT adsorbed to RCP and (**B**) covalently coupled BMP2 Azide. (**i**) 4 weeks, (**ii**) 6 weeks, (**iii**) 8 weeks, (**iv**) 10 weeks and (**v**) 12 weeks. The arrows indicate the subcutaneously formed ectopic bone.

**Figure 5:**
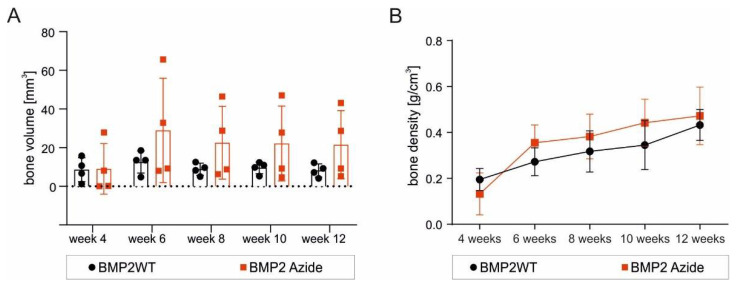
Evaluation of bone volume and bone density. Graphs show a micro-CT evaluation of (**A**) ectopic bone volume and (**B**) bone density over 12 weeks. *n* = 4. Statistical analysis was performed using non-parametric Mann–Whitney test. No significance was determined.

As the new BMP2 Azide variant covalently coupled to microspheres via SPAAC reaction showed promising results regarding specificity, efficacy and bioactivity in vitro, we next tested the RCP microspheres covalently functionalized with the new BMP2 Azide variant in a subcutaneous rat model. De novo formed bone was observed by micro-computed tomography (micro-CT) from week 4 onward using RCP microspheres with covalently coupled BMP2 Azide or with ad-/absorbed BMP2 WT (Figure 4), while sham controls using empty RCP microspheres revealed no bone formation (Figure S4). Four out of seven rats injected with BMP2 WT and BMP2 Azide functionalized microspheres showed growth of ectopic bone tissue. The bone volume reached a peak at week 6 and maintained a steady state for both BMP2 Azide and BMP2 WT functionalized RCP microspheres until 12 weeks post-implantation (Figure 5A). Bone density was increasing over time (Figure 5B). Both bone volume and bone density appeared slightly higher for the covalently coupled BMP2 Azide when compared to the control, albeit those differences reached no statistical significance.

### 2.5. The Morphology of the De-Novo-Induced Bone Tissue Highly Varies Depending on the Specific Immobilization Technique

**Figure 6:**
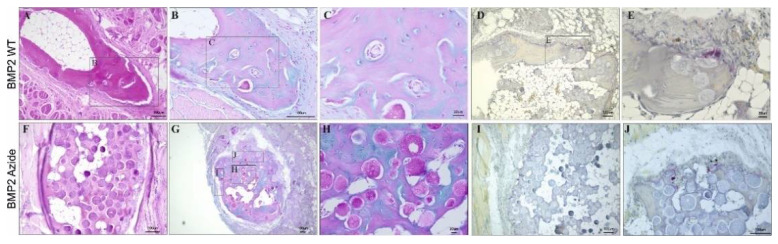
Histological evaluation of ectopic bone formation in BMP2 WT and BMP2 Azide RCP-based implants. Representative images of various staining techniques analyzing ectopic bone formation after 12 weeks of subcutaneous implantation in (**A**–**E**) BMP2 WT-induced ossicles and (**F**–**J**) BMP2 Azide-induced ossicles. Staining techniques: H&E (**A**,**F**), Alcian Blue, (**B**,**C**,**G**,**H**) tartrate-resistant acid phosphatase (TRAP) (**D**,**E**,**I**,**J**). Scale bar represents 100 µm (**A**,**B**,**D**,**F**,**G**,**I**,**J**), 20 µm (**C**,**E**,**H**).

Despite BMP2 Azide- or BMP2 WT-induced ossicles appearing similar by micro-CT analysis, relevant differences in the sub-structural organization of the formed ossicles were found (Figure 6). Ossicles formed by ad-/absorbed BMP2 WT revealed a nutshell-like bone structure, reminiscent of dense cortical bone, which was filled with fatty tissue in its interior (Figure 6A). Instead, the ossicles formed by covalently coupled BMP2 Azide variant revealed a more uniform, sponge-like structure, intermingled with RCP microspheres, still visible 12 weeks post-implantation (Figure 6F). In both conditions, H&E staining confirmed the presence of cortical bone with osteocytes in lacunae (Figure 6A,F), which are embedded in concentric layers of mineralized bone matrix (pink) and transient proteoglycan-rich, non-mineralized bone precursor tissue (blue) discriminated by Alcian Blue staining (Figure 6B,C,G,H). Additionally, TRAP^+^ cells are scattered along the external surface of the BMP2 WT- and BMP2 Azide-induced ossicles (Figure 6D,E,I,J). The TRAP staining is intended to identify osteoclasts. While it is not 100% specific for osteoclasts, large multinucleated TRAP-positive cells presenting along the newly formed bone is quite conclusive evidence for the presence of osteoclasts and, therefore, bone remodeling. Additionally, Figure S5 gives an overview of all BMP2 WT- and BMP2 Azide-induced ossicles, and it is possible to observe a substantial difference in the number of microspheres remaining at the end of the study between the two conditions. At 12 weeks post-implantation, only a few RCP microspheres were still visible in the different ossicles induced by BMP2 WT, while the number of microspheres in BMP2 Azide-induced ossicles appeared much higher.

## 3. Discussion

In this work, we show that BMP2 Azide covalently immobilized to a scaffold resulted in the formation of ectopic bone with a different morphology compared to that induced by ab-/adsorbed BMP2 WT. In contrast to commonly used immobilization techniques, our site-specific coupling allows the production of a scaffold with controllable and reproducible osteogenic properties. This is achieved by introducing the functional group at a specific site within the BMP2 WT structure, ensuring the preservation of the variant’s bioactivity and accessibility of receptor binding sites, thus avoiding steric hindrance after coupling to microspheres.

### 3.1. BMP2 Azide: A New BMP2 Variant Optimized for Site-Directed Coupling to Scaffolds

In previous studies, several BMP2 variants were created to improve the ligand´s bioactivity in vivo. BMP2 containing additional heparin/heparan sulfate binding domain showed enhanced osteogenic activity in vivo, possibly due to a stronger matrix binding, which results in a longer retention time at the site of action [39]. Other BMP2 variants, including additional cysteines, were created to enable coupling via the free sulfuryl (SH) group present in the side chain of this amino acid [21,34]. However, cysteines in BMPs are strongly required for the formation of intra- and inter-molecular disulfide bridges, which are crucial for structure stabilization and, thus, bioactivity. Therefore, the presence of an additional “free” cysteine residue might lead to the formation of multimers and improperly folded dimers [34]. Other immobilization methods include the incorporation of unnatural amino acids (UAA), such as those with alkyl- and azido-appended functionalities for click and Staudinger reactions, photo-reactive sidechains for crosslinking and intrinsically fluorescent coumaryl and dansyl moieties [32,40]. In our previous work, we used this technique and described the introduction of an artificial amino acid into BMP2 in detail [34]. In brief, a BMP2 variant harboring the artificial amino acid N-Propargyl-lysine (Plk) close to its N-terminal end (BMP2 K3Plk) was produced. The choice for the introduction of this UAA close to the exposed and flexible N-terminus was to facilitate protein expression, purification and refolding and, moreover, offering the best possible chance for coupling via CuAAC reactions. The uncoupled BMP2 K3Plk showed biological activities comparable to those of the wild-type protein. However, when this variant was coupled to bulky scaffolds, the interaction with the cell surface receptors was partially hampered due to steric hindrance [34]. Therefore, we tested different positions for the optimal introduction site of the artificial amino acid. From all tested variants, only the BMP2 variant in which glutamic acid 83 was replaced in each chain by H-L-Lys(EO-N_3_)-OH residue could be produced in high yields while maintaining structural and biological properties. Furthermore, the functional group at this position allowed a SPAAC reaction with high efficacy and maintaining bioactivity after coupling. Nonetheless, a loss of bioactivity was seen after coupling the BMP2 Azide to a bulky DBCO-functionalized fluorophore (DBCO-Cy5), probably due to masking of receptor-binding epitopes. This effect was significantly reduced by an introduced PEG_4_ spacer (DBCO-PEG_4_-5/6 Texas Red). However, the remaining inhibitory effect might be due to an unspecific interaction of the PEG moieties with the exposed amino acids on the ligand’s surface, which was also reported for other PEGylated proteins [41,42].

### 3.2. Induction of ALP Expression in C2C12 Cells by RCP with Covalently Coupled BMP2 Azide Is Restricted to Cells in Direct Contact

Specificity and efficacy of the SPAAC reaction were also proven after coupling the ligand to DBCO-functionalized microspheres. Quantitative analyses showed that in our release experiments, almost 100% of the covalently coupled BMP2 Azide was still bound to the used RCP even after two weeks of incubation in aqueous solution. In the case of only ab-/adsorbed proteins, −30% of the load of BMP2 Azide or WT was released at the same time. These values indicate improved retention characteristics of the collagen-based biomaterial. By tuning pore and/or particle sizes, crosslinking times and the applied crosslinking methods, these microspheres showed a lower initial burst release kinetics than other systems [43]. The cell-based experiments also demonstrated that the retained proteins were, in all cases, biologically active, since the loaded RCP microspheres induced ALP expression in C2C12 cells even when the protein was covalently coupled. In the latter scenario, ALP expression was restricted to cells being in direct contact with the BMP2 Azide-functionalized RCP spheres, implying that the protein was indeed covalently coupled and not just absorbed. Thus, these results confirm those of our previous study, showing that covalently coupled BMP ligands exert the same biological function in C2C12 cells as the unbound, soluble ligand [34]. Important to note is that the BMP2 variant used by Tabisz et al. was coupled to agarose beads at their N-terminus, also by a flexible (PEG_4_) linker [34]. This approach was not optimal but could partially allow a correct orientation toward the receptors on the cell surface. In this study, the BMP2 Azide was coupled via a similar linker, but the position of the integrated H-L-Lys(EO-N3)-OH residue allowed a correct alignment of the immobilized ligand toward cell surface receptors, as shown in Figure 1A. However, both studies clearly showed that an internalization of the ligand, as it is described by Knaus and co-workers [44,45], does not seem to be necessary at least for the induction of ALP activity in this cell-based assay. Thus, neither the characteristics of the used material nor the known biological activities of the used BMP2 variant could explain the remarkable differences in the morphology and structure of the ectopically formed bone in our animal experiment. These results clearly demonstrated that the bone-inducing activity of both BMP2 proteins, BMP2 WT and BMP2 Azide, is sufficient to induce the formation of ectopic bone but that the morphology of the formed ossicles strongly depends on the specific delivery method.

### 3.3. The Delivery Methods Significantly Affect the Morphology of the Ectopically Formed Bone

It can be assumed that the structural differences in the formed bone that were observed when comparing BMP2 WT and BMP2 Azide depend on different “release kinetics” significantly influencing cell recruitment and/or cell differentiation. The ab-/adsorbed protein simply seems to be released from the implant toward the subcutaneous environment by a diffusion-like process, which most likely promotes the formation of a dense, spherical cortical bone shell at the periphery and leaving abundant adipose tissue inside. Instead, the covalently coupled BMP2 Azide is not diffusible, and a release of this protein can only occur upon degradation of the RCP microspheres, since a proteolytic cleavage of the used linker (PEG_4_) by proteases such as, e.g., matrix metalloproteases, is unlikely. However, microspheres were still detected after 12 weeks inside the BMP2 Azide-induced ossicles. This might indicate that the covalently coupled protein provokes adherence and differentiation of responsive cells to the functionalized surface of the RCP microspheres, where bone tissue emerges around the microspheres. It is relevant to note that the covalent coupling of BMP2 prevents the formation of a BMP2 gradient, which is deemed important for cell recruitment. Osteoclast activity is visualized by TRAP-positive staining, which was located along the edge of newly formed bone, indicating active bone remodeling [46,47].

## 4. Materials and Methods

### 4.1. Cloning, Expression and Purification of BMP2 Azide

The BMP2 Azide was generated by site-directed mutagenesis using the Rapid-PCR methodology from Costa and Weiner [31]. The codon encoding for Glu83 (GAA) (mature part) of the human BMP2 sequence was replaced by the amber stop-codon TAG. The resulting coding sequence (BMP2-E83stop) was subcloned into the bacterial expression vector pET11a-pyltRNA (kind gift of M. Rubini; Konstanz, Germany) and verified by DNA sequencing. Expression and purification of BMP2 Azide were performed as described previously for other BMP2 variants [34,35,37,48]. Briefly, BL21(DE3) one-shot bacteria were co-transfected with pET11a-pyrtRNA-BMP2 Azide and pRSFduet-pyltRNAsynth, which encodes for the corresponding pyrrolysyl-tRNA synthetase. A single colony was inoculated overnight in lysogeny broth (LB) and propagated in terrific broth (TB). At OD_600_ = 0.5, 15 mM H-L-Lys(EO-N_3_)-OH (Azide) was added, and protein expression was induced by adding IPTG at a 1 mM concentration. Detailed protocols of BMP2 expression, inclusion body recovery and protein purification, are described in Siverino et al. 2018 [48].

### 4.2. Analyses of BMP2 Azide

#### 4.2.1. SDS Gel Electrophoresis and Coomassie Brilliant Blue Staining

BMP2 WT and BMP2 Azide (5 µg) were separated by SDS-PAGE Electrophoresis under non-reducing or reducing conditions and afterward stained with Coomassie Brilliant Blue R-250 (Thermo Fisher Scientific, Waltham, MA, USA).

#### 4.2.2. Alkaline Phosphatase Assay

The ALP assay was performed using the myoblastic cell line C2C12 (ATCC CRL-172) as previously described [49]. Cells were treated with different concentration of soluble BMP2 WT or BMP2 Azide, and ALP expression was analyzed after 72 h of incubation. For ALP detection by a spectrophotometric assay, cells were lysed, and a paranitrophenyl-phosphate (pNPP) solution (2 mg/mL of paranitrophenyl-phosphate) was added. Absorption at 405 nm was measured using a Tecan infinite M200 (Tecan) reader. Dose–response curves were generated using the software OriginPro9.1. ALP staining was performed using 1-Step™ nitro-blue tetrazolium chloride/5-bromo-4-chloro-3′-indolyphosphate ptoluidine salt (NBT/BCIP) substrate solution (Thermo Fisher Scientific, Waltham, MA, USA).

#### 4.2.3. Surface Plasmon Resonance (SPR) Spectroscopy

A Reichert4SPR surface plasmon resonance system (Reichert Technologies, Buffalo, NY, USA) was used for all SPR measurements. Measurements were performed at ambient temperature (25 °C) using 10 mM HEPES pH 7.4, 500 mM NaCl, 3.4 mM EDTA and 0.005% (*v*/*v*) Tween-20 as running buffer. The flow rate for interaction data acquisition was set to 10 μL/min. For the interaction analysis of BMP2 WT and the variant BMP2 Azide with the extracellular domains of BMPR-IA or ActR-IIB, the receptor ectodomains were biotinylated at a 1:1 molar stoichiometric ratio using Sulfo-NHS-LC-biotin (Pierce, Thermo Fisher Scientific, Waltham, MA, USA) according to the manufacturer’s recommendations. A CMD200 biosensor chip (Xantec Bioanalytics GmbH, Düsseldorf, Germany) was first activated using EDC/NHS according to manufacturer’s recommendation; then, streptavidin was perfused over the activated sensor surface at a concentration of 100 μg/mL, thus immobilizing streptavidin corresponding to 2000 to 2500 resonance units (RU) per channel. The biotinylated receptor ectodomains were subsequently immobilized onto this streptavidin sensor surface at a density of approximately 350 to 500 RU. For binding kinetic analyses, six different analyte concentrations starting at 160 nM (log 2 dilution series) were used. The association time was set to 360 s. Dissociation data were obtained from perfusion with running buffer for 180 s. After each ligand perfusion, the sensor chip was regenerated by perfusing 20 s a regeneration solution consisting of 6 M Urea/Acetic acid pH 3. To remove bulk face effects (buffer jumps, etc.) and unspecific binding to the chip matrix, the interaction of the analyte to the unmodified streptavidin surface (empty channel 4) was subtracted from all binding data. Binding affinities were calculated by fitting the association and dissociation phase of the sensorgrams using a grouped regression analysis of the rate constants and employing 1:1 Langmuir-type interaction model (global fit). Standard deviation of equilibrium binding constants was derived from two independent experiments using six different analyte concentrations.

### 4.3. Coupling of BMP2 Azide by Strain-Promoted Azide–Alkyne Cycloaddition (SPAAC) to a Dibenzocyclooctyne (DBCO)-Functionalized Fluorophore

An amount of 20 μM of BMP2 Azide was incubated for 120 min with different concentrations (1 mM, 100 µM, 10 µM, 1 µM, 0.1 µM, 0.01 µM and 0 µM) of a DBCO-functionalized fluorophore (DBCO-Sulfo-Cy5, Jena Bioscience, Jena, Germany) in a total volume of 50 µL, while BMP2 WT was used as negative control. Samples were separated by SDS-PAGE Electrophoresis, and gels were analyzed under Cy5 detection channel (imaging system FluorChemQ, Biozym Scientific GmbH, Hessisch Oldendorf, Germany and the AlphaView 3.2.2.0 software, Cell Biosciences, Santa Clara, CA, USA) and protein quantity verified by Coomassie Brilliant Blue. BMP2 Azide or BMP2 WT (20 µM) were reacted to 100 µM DBCO-PEG_4_-5/6-Texas Red (Jena Bioscience) for different reaction times (15, 30, 60, 120 min or overnight) and applied to C2C12 cells. Alkaline phosphatase expression was measured by absorbance, as described above. For the ALP staining, 20µM of BMP2 Azide and 100 µM DBCO-PEG_4_-5/6-Texas Red were reacted for 2 h at room temperature in a total volume of 50 µL. As control, BMP2 Azide was incubated with milliQ water in the same reaction volume as the BMP2 Azide reacted with DBCO-PEG_4_-5/6-Texas Red. Afterward, 200 nM of BMP2 Azide or BMP2 Azide-DBCO-PEG_4_-5/6-Texas Red were applied to C2C12 and incubated for 3 days at 37 °C, 5% CO_2_. After 3 days, media was removed, and ALP staining solution NBT/BCIP was applied to the cells, and images were taken 5 min after the application of the NBT/BCIP solution.

### 4.4. Coupling of BMP2 Azide to DBCO-Functionalized Microspheres

For the coupling reaction of BMP2 Azide, recombinant collagen-like peptide (RCP) microspheres produced from Fujifilm (Cellnest™, Fujifilm, Tilburg, The Netherlands) were used [50,51,52]. RCP production was performed at Fujifilm Manufacturing Europe (Tilburg, The Netherland), and microsphere production was described in Mumcuoglu et al. 2018 [43]. RCP microspheres were functionalized with 4 mM NHS-PEG_4_-DBCO linker (Jena Bioscence) in 1000 µL 0.1 M NaHCO_3_ buffer (pH 7.4). BMP2 Azide (20 µg) was incubated with DBCO-functionalized microspheres for 2 h and, as control, non-functionalized RCP microspheres were incubated with 20 µg of BMP2 Azide or BMP2 WT. After the reaction, the supernatant was collected, and samples were washed 3 times with PBS. All the supernatants from these washes were collected. Samples were then incubated in PBS for 2 weeks on a rocking platform, and the supernatants containing the protein released over 2 weeks were collected. All supernatants were used for the quantification analyses. Coupled or absorbed microspheres were stored at 4 °C upon application.

### 4.5. Quantification of Immobilized BMP2 Azide

The amount of protein immobilized to the microspheres was indirectly determined by quantifying the unbound BMP2 in the supernatant of the reaction by ELISA (PeproTech, Rocky Hill, NJ, USA). After SPAAC reaction or absorption, the supernatants were collected and analyzed by ELISA following the manufacturer’s protocol. The standard curves were performed using BMP2 WT and BMP2 Azide.

### 4.6. Injection of a Paste Containing BMP2-Functionalized Microspheres in a Subcutaneous Rat Model

Nine-week-old male Sprague Dawley (SD) rats (Charles River) were used in these studies. The animals were randomly assigned and housed in pairs in a specific pathogen-free (SPF) facility and allowed to adapt to the conditions of the animal house for 7 days before experimentation. The animals were maintained at 22 ± 5 °C on a 12 h dark/light cycle with ad libitum access to standard rat chow and water. The ARRIVE guidelines for animal experiments were followed. Micro-CT was performed every 2 weeks from week 2 onward. At 12 weeks after implantation, animals were euthanized with CO_2_, and specimens were harvested for ex vivo micro-CT analysis and histology.

An amount of 10 mg of RCP DBCO-functionalized microspheres was covalently coupled with 10 µg BMP2 Azide, or non-functionalized RCP (10 mg) were absorbed with 10 µg BMP2 WT. As sham control, non-functionalized microspheres without either absorbed or covalently coupled proteins were used. BMP2 Azide- or BMP2 WT-loaded microspheres (10 mg) were mixed with 30 mg of non-functionalized RCP. A paste was created by mixing the RCP microspheres with saline and then transferred to a 1 mL syringe, and 100 µL was subcutaneously injected in the dorsal part of the rat using a 19G needle (*n* =7 per condition). Each rat received 5 injections in 5 different subcutaneous pockets.

### 4.7. Micro-Computed Tomography (Micro-CT) Analysis

A Quantum FX micro-CT (Perkin Elmer, Waltham, MA, USA) was used to image the animals every two weeks until the end of the experiment. To image ectopic bone in vivo, the following parameters were used: Field of view: 73 mm, Voltage: 90 kV, Current: 160 μA, Scan Time: 120 s. To image the implants ex vivo, a field of view of 20 mm was used (other parameters were maintained).

### 4.8. Evaluation of Bone Density and Bone Volume

Trabecular and cortical bone mineral density (BMD) was measured based on calibration scanning, using two phantoms with known density (0.25 and 0.75 g/cm^3^) which were scanned under identical conditions using a Bruker Micro-CT (Bruker Corporation, Billerica, MA, USA). For image processing, the Analyze 11.0 software (Mayoclinic, Rochester, MN, USA) was used, and threshold levels were set to 0.13 g/cm^3^ in vivo and to 0.15 g/cm^3^ ex vivo (threshold levels were set to 0.11 g/cm^3^, 400 Hounsfield units). Bone density and bone volume were calculated as the mean of the *n* = 4 samples (BMP2 WT and BMP2 Azide) that formed bone, excluding the animals that did not show bone formation (zero values).

### 4.9. Histological Analysis of Explanted Implants

For histological analyses, samples were fixed in a 4% formalin solution and decalcified using 10% EDTA for 4 weeks. Implants were dehydrated and embedded in paraffin. The 5 µm thick sections were prepared and stained according to the standard procedures for hematoxylin and eosin (H&E), Alcian Blue and tartrate-resistant acid phosphatase (TRAP) staining (Acid Phosphatase, Leukocyte (TRAP) Kit, Sigma-Aldrich, (St. Louis, MO, USA).

### 4.10. Statistics

Statistics were performed using unpaired t-test for the SPR data; two-way ANOVA–Dunnett’s multiple comparisons test for the ALP assays to compare BMP2 Azide to BMP2 Azide reacted with the fluorophore for different reaction times; a non-parametric Mann–Whitney test for the comparison of bone volume and bone density between the two different groups (BMP2 Azide and BMP2 WT); and ordinary one-way ANOVA, Tukey’s test for the quantification of the released proteins by ELISA.

### 4.11. Study Approval

All animal experiments were performed with prior approval of the ethics committee for laboratory animal use (protocol number EMC 15-114-05) at Erasmus Medical Center, Rotterdam, The Netherlands. Nine-week-old male Sprague Dawley (SD) rats (Charles River) were used in these studies. The ARRIVE guidelines for animal experiments were followed.

## 5. Conclusions

The profound morphological differences between ab/adsorbed vs. covalently coupled proteins were solely observed in an ectopic model. Therefore, the next step would require the application of the covalently coupled protein in a long-bone-defect animal model to address, in parallel, inflammation and angiogenesis at different time points. Before proceeding with further animal testing, a scaffold incorporating the required functional sites for the coupling chemistry should be designed. This would avoid unnecessary expenses incurred in the integration of required linkers, and more importantly, would reduce the use of synthetic structures, which might provoke unwanted immune reactions.

Our work presented here clearly showed that a covalently coupled osteogenic growth factor is capable of inducing bone formation in vivo. The diffusion of the growth factor is abolished, thus eliminating the severe side effects often observed in the clinics after implantation of the commercially available products. Hence, our approach suggests that the amount of protein to be injected most likely represents the minimal effective dose required to induce bone formation. An optimized delivery system employing covalently coupled BMP2 might lead to a reduction in the required protein doses and, consequently, also to reduced costs and fewer side effects. Therefore, the technique developed in this study opens the field for several new translational applications in regenerative orthopedics.

## Data Availability

Not applicable.

## Supplementary Materials

The following supporting information can be downloaded at: https://www.mdpi.com/article/10.3390/ijms23073928/s1.
