# Supplementary material for: Site-Directed Immobilization of an Engineered Bone Morphogenetic Protein 2 (BMP2) Variant to Collagen-Based Microspheres Induces Bone Formation In Vivo
Source: Int J Mol Sci. 2022 Apr 1;23(7):3928. doi: 10.3390/ijms23073928 (PMC8999711; doi:10.3390/ijms23073928)
Supplement: Supplementary file 1 [file ijms-23-03928-s001.zip › ijms-1626442-Supplementary Materials.pdf]

Supplementary Materials:

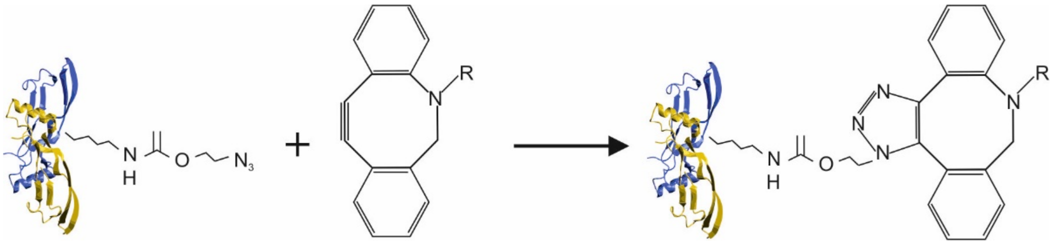

Figure S1: Reaction of BMP2 Azide to a DBCO moiety. The azide group of the BMP2 Azide reacts with a dibenzylcyclooctyne (DBCO) moiety creating a triazole.

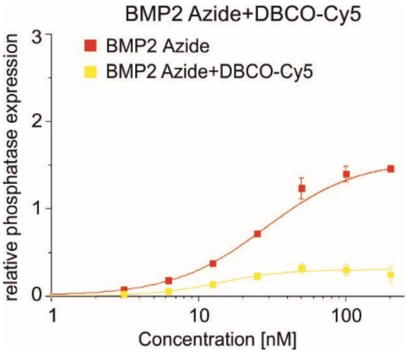

Figure S2: Evaluation of incubation of BMP2 Azide and DBCO-Cy5. C2C12 cells were exposed to BMP2 Azide which was before incubated with DBCO-Cy5 for 120 min. Untreated BMP2 Azide was used as control. Statistical analyses of the ALP data were performed using 2-way ANOVA- Sidak's multiple comparisons test. Significant differences were observed by comparing the ALP activities induced by uncoupled BMP2 Azide and those induced by BMP2 Azide + DBCO-Cy5 from 6.25nM until 200 nM concentrations; $p < 0.0001$.

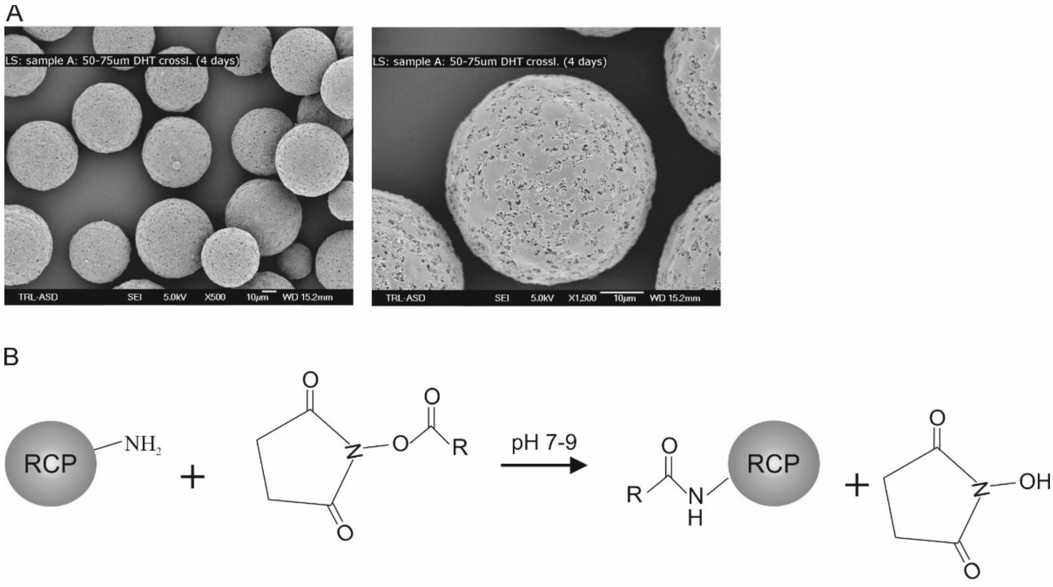

Microsphere type	Average free amino group ±SD (nmol/100g)
RCP 50-70 µm (high) HMDIC	14.6 ± 0.5
RCP 50-70 µm (low) HMDIC	28.0 ± 4.8
RCP 50-70 µm DHT 4 days	41.4 ± 2.1

Figure S3: Characterization of recombinant collagen like peptide (RCP) microspheres (Cellnest™). (A) Scanning Electron Microscopy (SEM) images of RCP microspheres DHT crosslinked (4 days) with average size of 50 – 75 µm. (B) Depiction of the reaction of a free amine (NH_2) group within the RCP microsphere with an NHS functionalized linker (R: PEG_4-DBCO). Table of residual free amines on different microspheres using a colorimetric assay, based on the incorporation of 2,4,6-trinitrobenzenesulfonic acid (TNBS) (43). Based on the amounts of residual free amine groups, DHT crosslinked microspheres with a 50-75 µm diameter range were chosen for the functionalization with the NHS-PEG_4-DBCO linkers to maximize the functionalization rate. The production of microspheres, measurements of free amine and SEM were performed at Fujifilm Manufacturing Europe (Tilburg, The Netherland).

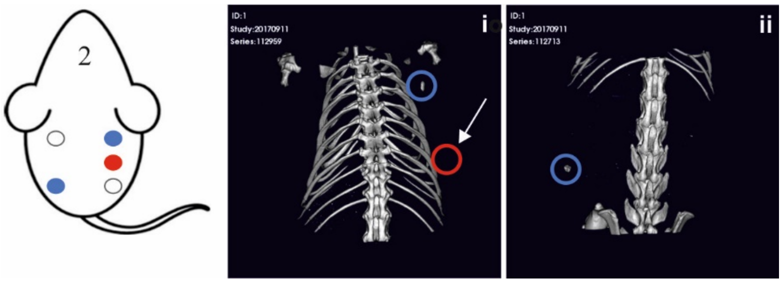

Figure S4: Micro-CT scan at 12 weeks of negative control. The left scheme shows the application layout for BMP2 WT ad/absorbed RCP microspheres(blue dots) and a negative control (empty microspheres, red dot). Micro-CT images ((i) upper and (ii) lower scan of the back of the rat show that at 12 weeks calcified tissue can be observed at positions where BMP2 WT adsorbed RCP (blue circles) have been implanted, while no calcification was detected for the negative control (empty RCP microspheres, white arrow and red circle).

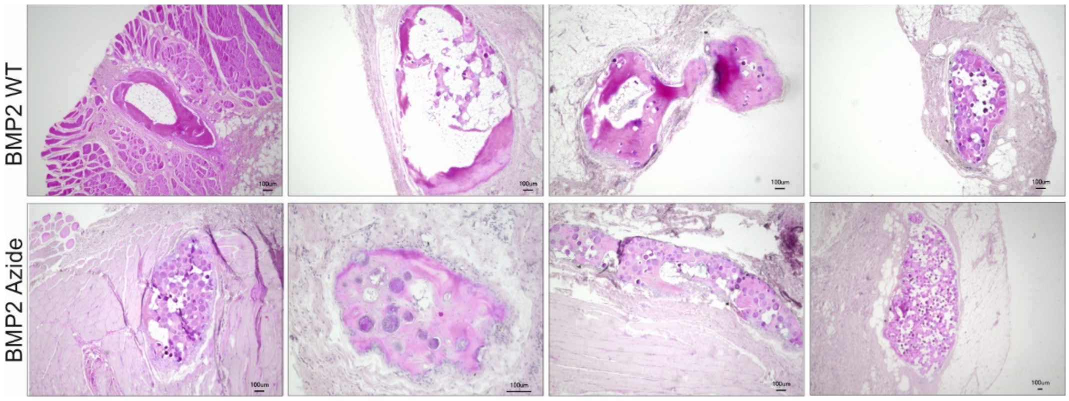

Figure S5: Histological comparison of ectopic bone formation between BMP2 WT and BMP2 Azide. H&E staining of the retrieved implants at 12 weeks after implantation. Upper panel shows the BMP2 WT-induced ossicles, while the lower panel represents the BMP2 Azide-induced ossicles.
